# Sex-specific modification of progesterone receptor expression by 17β-oestradiol in human cardiac tissues

**DOI:** 10.1186/2042-6410-1-2

**Published:** 2010-11-04

**Authors:** Georgios Kararigas, Eva Becher, Shokoufeh Mahmoodzadeh, Christoph Knosalla, Roland Hetzer, Vera Regitz-Zagrosek

**Affiliations:** 1 Institute of Gender in Medicine, Charite Medical University, Hessische Strasse 3-4, 10115 Berlin, Germany; 2 Centre for Cardiovascular Research, Charite Medical University, Hessische Strasse 3-4, 10115 Berlin, Germany; 3 German Heart Institute Berlin, Augustenburger Platz 1, 13353 Berlin, Germany

## Abstract

**Background:**

Although circulating levels of sexual hormones in elderly men and women are low and quite similar, the adaptation of the elderly heart to stress differs between the sexes. We have hypothesized that the effects of sexual hormones in the heart may differ in men and women. Here, we assessed whether 17β-oestradiol regulates gene expression in the human heart in a sex-dependent manner. We selected the progesterone receptor as a well studied 17β-oestradiol target that may be pathologically linked to cardiac remodelling.

**Methods:**

In order to assess the *ex vivo *effects of 17β-oestradiol in intact human cardiac tissues, we developed a 24-h model for the culture of human atrial myocardium. We verified tissue viability after 24 h in culture with two standard assays to determine the degree of apoptosis and metabolic activity of cardiac tissues. Progesterone receptor mRNA and protein level were measured after 24-h treatment of tissues with 17β-oestradiol. Statistical analysis was performed by the Mann-Whitney *U *test and two-way ANOVA.

**Results:**

We established a tissue culture model that allows for the study of viable human cardiac tissue over a 24-h period. After 24 h, cultured cardiac tissues revealed low apoptosis, retained their metabolic activity and, therefore, remained viable. Treatment with 17β-oestradiol led to an induction of the progesterone receptor mRNA level in female (*P *= 0.001) but not in male tissues. Similarly, there was an increase in the level of progesterone receptor protein in female tissues (*P *= 0.03), while a decreasing trend was observed in male tissues (*P *= 0.079) exposed to 17β-oestradiol.

**Conclusions:**

Our novel finding may offer a molecular explanation for the sex-specific differences observed in cardiac remodelling. The culture model we established for human cardiac tissue will facilitate the study of cellular processes in health and disease and will be of use for pharmacological testing.

## Background

Human and animal studies have demonstrated sex-specific differences in cardiac remodelling, where females have a better outcome than males. However, the molecular mechanisms resulting in males undergoing more severe remodelling than females are poorly understood. In the hypertrophic response, cardiac remodelling occurs as a result of abnormal fibroblast proliferation, which leads to fibrosis. The steroid hormone 17β-oestradiol (E2) is largely considered to contribute significantly to the manifestation of sex-specific differences. Indeed, multiple effects for E2 on the cardiovascular system have been reported [1]. Along this line, E2 is expected to limit fibrotic remodelling [2,3] mediated by less well described mechanisms.

E2 regulates a large number of genes, including progesterone receptor (*PGR*). The PGR protein belongs to the superfamily of ligand-activated transcription factors that regulate gene expression and mediates the genomic and non-genomic actions of progestins. It is expressed and functionally active in a number of different cell types, including vascular smooth muscle cells [4], endothelial cells [5], cardiac fibroblasts [6] and myocytes [7]. To date, E2 has been shown to regulate the *PGR *gene in a sex-dependent fashion in specific brain regions of adult rats [8] and in human lung adenocarcinoma cell lines [9].

The study of the effects exerted by E2 directly on cells of the heart is crucial. Nevertheless, although conventional two-dimensional cell culture models have been extremely useful for studying extra- and intra-cellular physiology, some hypotheses do not lend themselves to two-dimensional models [10]. For instance, the microenvironment that defines the niche, a dynamic architecture with anatomic and functional dimensions [11], may not be realized in conventional cell culture systems. Furthermore, tissues are made up of more than a single cell type and the interaction of cells drives their response to stimuli. As a result, hormones may exert a different effect on the same cell type at a tissue or organ level in comparison to a cell culture system [12]. Generally, such systems also lack the influence of a normal extracellular matrix (ECM) and even less that of an abnormal ECM, as occurs in target-organ damage such as interstitial fibrosis in the heart which contributes to cardiac remodelling.

To date, due to obvious hurdles, there have been no reports of long-term gene and protein expression studies addressing the direct effects of stimuli and investigating the underlying molecular mechanisms of physiological and pathological cellular processes in the heart of humans. Murine embryonic heart slices [13] and isolated mammalian papillary muscle strips [14,15] have only allowed the measurement of the development of mechanical force in a narrow temporal space. Isolated myocardial strips have also been used for a short stimulation period [16], thus making longer-term molecular studies of biological processes difficult and resulting in imperfectly defined viability.

In the present study, we describe an *ex vivo *tissue culture system which maintains healthy human cardiac tissues in culture for up to 24 h. We assured viability of the cultured tissues under defined conditions. Subsequently, we applied this model to treat cardiac tissues with E2. We provide the first evidence that E2 induces the expression of the PGR messenger RNA (mRNA) and protein in the heart tissues of women but not in those of men. This finding contributes to a molecular explanation for the sex-specific differences observed in the diverse remodelling responses in men and women and in animal studies after myocardial infarction. It could also have major implications for hormone therapy.

## Methods

### Patients and tissue harvest

All chemicals and reagents were purchased with highest purity grade available from Sigma-Aldrich (MS, USA) unless otherwise indicated. The study was approved by the Charite Medical University Berlin Ethics Committee and complies with the principles outlined in the Declaration of Helsinki. Written consent from patients undergoing coronary bypass surgery was obtained for use of their tissue from the right atrium, ensuring that the tissue sample collection would originate from a healthy part of the heart. Tissues were collected from 15 men and six women for the whole study and subsets of these were used for different assays. There were no significant differences in the medication between males and females and their baseline characteristics are shown in Table 1. A piece of the appendage of the right atrium measuring about 10 mm long was excised just before cardiopulmonary bypass. Tissues were quickly transported from the operating room to the laboratory in cold Krebs Henseleit buffer (KHB) (120 mM NaCl, 5 mM KCl, 2 mM MgSO_4_, 1.2 mM NaH_2_PO_4_, 20 mM NaHCO_3_, 10 mM glucose, 0.25 mM CaCl_2_, pH 7.4).

**Table 1:** Characteristics of the individuals whose tissues were employed in the study.

	Men (*n *= 15)	Women (*n *= 6)
Age (years) (mean ± SD)	65.07 ± 6.43	76.00 ± 3.74
BMI (kg/m^2^) (mean ± SD)	27.47 ± 3.26	23.50 ± 3.35
EF (%) (mean ± SD)	59.80 ± 5.99	54.33 ± 8.81

BMI, body mass index; EF, ejection fraction; SD, standard deviation.

### Tissue culture and tissue treatment

Upon tissue removal and immediate incubation in cold KHB, all tissues were processed within 20 min. Prior to collecting the tissue, the KHB was freshly oxygenated with carbogen gas (95% O_2_, 5% CO_2_) for 1 h at a speed of 0.5 L/min achieving an O_2 _pressure of 247.9 ± 8.3 mm Hg on average (Rapidlab 865; Bayer Diagnostics/Chiron Diagnostics, Leverkusen, Germany). The collected tissue was examined macroscopically and any fat detected was removed. The tissue was cut further into uniformly smaller pieces about 3-5 mm long each depending on the size of the initial sample. Then, each smaller piece was transferred to a 50 mL Falcon tube containing a modified KHB supplemented with 20 mM 2,3-butanedione monoxime and 10^-8 ^M 2-hydroxypropyl-β-cyclodextrin (HBC)-encapsulated E2 or 10^-8 ^M HBC (control for E2 treatment) and was incubated at 37°C in a conventional water bath for 24 h. This concentration was chosen to obtain an E2 level of physiological range. Following the completion of the treatment, the tissues were shock frozen in liquid nitrogen and stored at -80°C until further analysis. For the purpose of testing the viability of the tissues, the following groups with three tissues from three individuals each were investigated: fresh (control for viability; shock frozen in liquid nitrogen or incubated in a KHB-MTT solution immediately after cutting), damaged (control for death; incubated in distilled water at room temperature for 24 h), E2-treated (cultured and treated with E2 for 24 h) and HBC-treated (control for E2-treatment; cultured and treated with HBC for 24 h).

### Terminal deoxynucleotidyl transferase (TdT) assay

A common method for the detection of apoptosis was applied in order to establish the degree of cell death in the different tissue groups. The Fluorescein Fragel™ DNA Fragmentation Detection Kit (Calbiochem; VWR, Darmstadt, Germany,) was used according to the manufacturer's instructions in 5-μm-thick slices from the core of frozen tissues embedded in Neg-50 frozen section medium (Thermo Scientific, Richard-Allan Scientific, MI, USA). Total and apoptotic nuclei were determined by a fluorescence microscope (Leica DMIREZ; Leica Microsystems, Wetzlar, Germany) using a Retiga 1300 camera (Q Imaging) and Openlab version 5.5.0 (Improvision). To calculate the apoptotic index, five images of different areas of a tissue section covering most of the section were taken from three independent tissues for each group. Total and apoptotic nuclei were counted and the number of apoptotic nuclei was divided by the total number of all nuclei in each of the five images. Then, the mean of these for each section were taken.

### 3-(4,5-dimethylthiazol-2-yl)-2,5-diphenyltetrazolium bromide (MTT) assay

In order to assess the metabolic activity of the cells, we used a modified colorimetric MTT assay. This assay is based on the enzymatic activity of the mitochondrial dehydrogenase, which converts the soluble yellow MTT salt into insoluble purple formazan crystals by cleaving the tetrazolium ring [17]. Tissues were incubated in 1 mL KHB supplemented with 100 μL MTT solution [5 mg/mL in phosphate buffered saline (PBS)] at 37°C for 3 h. Following incubation, tissues were either frozen in liquid nitrogen or incubated in 400 μL lysis buffer (99.4 mL DMSO, 0.6 ml acetic acid glacial 100%, and 10 g sodium dodecyl sulphate) at 4°C overnight in order to dissolve the formazan crystals formed within the cells. Then, 100 μL of the lysed solution were transferred to a 96-well plate and the absorbance was measured at 570 nm on a microtitre plate spectrophotometer (Benchmark Plus; Bio-Rad, CA, USA). In order to examine formazan formation following MTT exposure, frozen tissues were embedded in Neg-50 frozen section medium and serial cross-sectioned. Five micrometre thick slices from the tissue core were mounted on slides and were examined microscopically (Leica DMIREZ; Leica Microsystems). Images were taken using a Retiga 1300 camera (Q Imaging) and Openlab version 5.5.0 (Improvision).

### Gene expression study

Total RNA was isolated from nine male and four female frozen tissues using the Rneasy Fibrous Tissue Mini kit (Qiagen, Hilden, Germany) following the manufacturer's protocol. The RNA quality and quantity was established using a 2100 Bioanalyzer (Agilent Technologies, CA, USA). Then, 0.5 μg of total RNA was reverse transcribed into cDNA using the High-Capacity cDNA Reverse Transcription kit (Applied Biosystems, CA, USA) according to the manufacturer's instructions. Quantitative real-time RT-PCR reactions were performed in 25 μL final volume using SYBR Green (Applied Biosystems) in a 7000 ABI Prism Instrument (Applied Biosystems). Reactions where RNA or reverse transcriptase had previously been omitted during reverse transcription were used as negative controls. In order to normalize cDNA concentration in different samples, the expression of pyruvate dehydrogenase (lipoamide) beta (*PDHB*), hypoxanthine phosphoribosyltransferase 1 (*HPRT1*) and ribosomal protein large P0 (*RPLP0*) was measured and a normalization factor from the mean of their expression levels was calculated, by which the quantity of *PGR *was divided. The sequences of the primer pairs used and the size of the products are: *HPRT1*: 5'-CTTTGCTGACCTGCTGGATT-3' and 5'-TATGTCCCCTGTTGACTGGT-3', 121 bp; *PDHB*: 5'-GGTATGGATGAGGAGCTGGA-3' and 5'-CTTCCACAGCCCTCGACTAA-3', 102 bp; *PGR*: 5'-TCCTTACCTGTGGGAGCTGT-3' and 5'-CGATGCAGTCATTTCTTCCA-3', 90 bp; and *RPLP0*: 5'-ACGGGTACAAACGAGTCCTG-3' and 5'-AGCCACAAAGGCAGATGGAT-3', 104 bp.

### Protein abundance analysis

Four male and four female tissues were homogenized in a detergent-containing lysis buffer with protease inhibitor. Per lane, 7.5 μg protein were loaded on 6% SDS-PAGE gels and transferred to nitrocellulose membranes. Antibodies against PGR (Cell Signaling) and actin (Santa Cruz, CA, USA; loading control) were used. Immunoreactive proteins were detected using the ECL Plus (GE Healthcare, Buckinghamshire, UK) and quantified by the ImageJ 1.41 version software.

### Statistical analysis

All experiments were performed with three or more independent tissue samples. Each tissue originated from a different individual. Data are shown as the mean ± standard deviation. All data were analyzed statistically using the R version 2.8.0 software. For the apoptotic (TdT) and the biochemical (MTT) assays, statistical comparisons between two groups were made using the one-tailed Mann-Whitney *U *test. For the gene and protein expression studies, normality of the data was evaluated by means of the Shapiro-Wilk test. Statistical analysis was performed using two-way ANOVA. All pairs of means were compared by Tukey's *post hoc *test adjusting for multiple comparisons. A value of *P *≤ 0.05 was considered statistically significant.

## Results

### Level of apoptosis in cultured tissues

**Figure 1:**
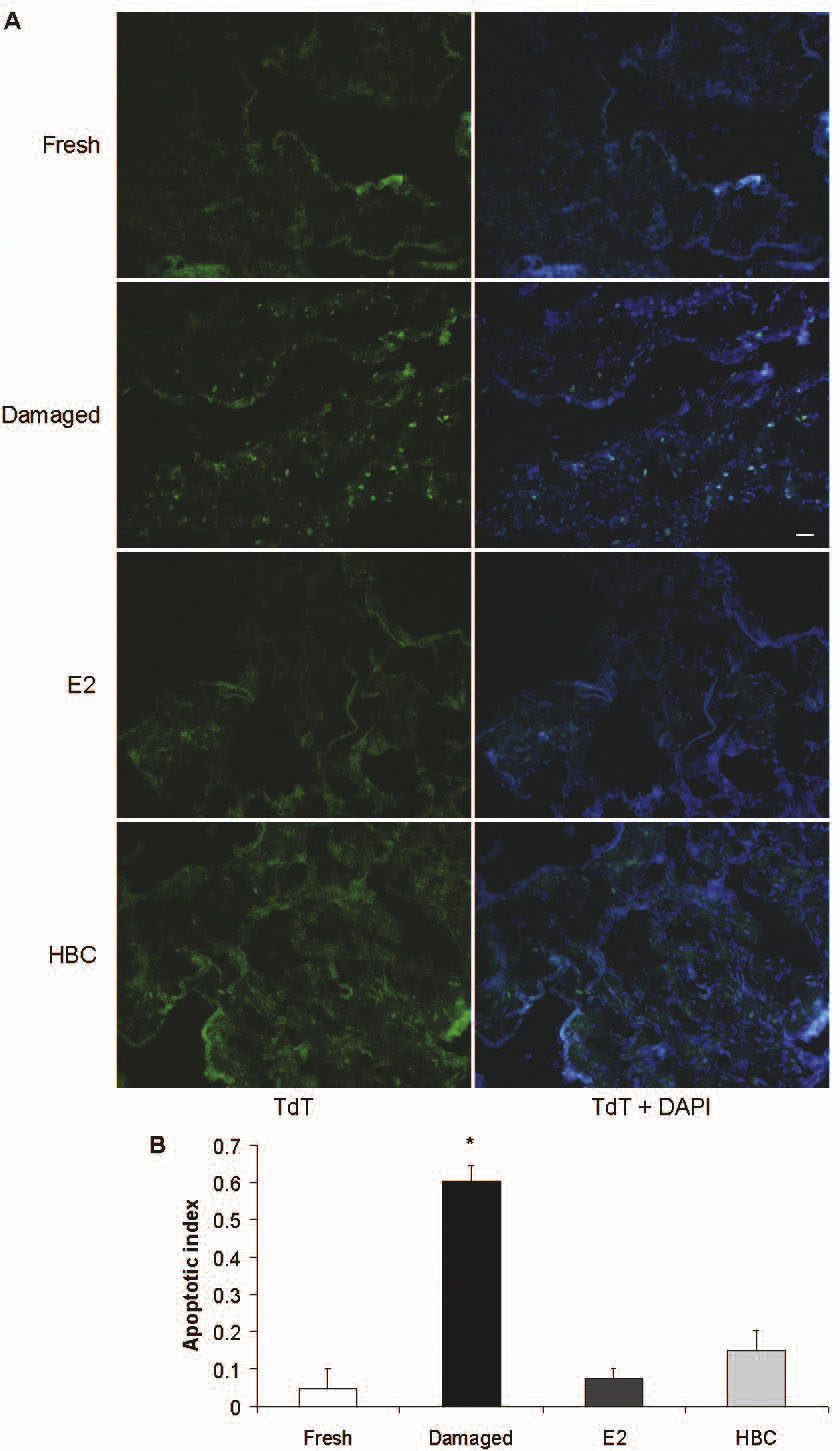
**Degree of apoptosis in cultured tissues**. (A) Representative images of 5 μm-thick tissue sections labelled with terminal deoxynucleotdyl transferase (TdT). Few apoptotic nuclei are present in E2- and 2-hydroxypropyl-β-cyclodextrin (HBC)-treated and fresh tissues, while large numbers of apoptotic-positive nuclei are observed in damaged tissues. Fresh: control for viability; damaged: control for death; HBC-treated: control for E2 treatment. TdT (green) indicates apoptotic nuclei. Merged images with DAPI are shown in right panels. Scale bar, 100 μm. (B) The apoptotic index is low in E2- and HBC-treated and fresh tissues but significantly higher in damaged tissues (*n *= 3, *P = 0.05 for damaged versus E2- and HBC-treated and fresh). Data represent means ± standard deviation.

Programmed cell death was assessed with the TdT assay. In apoptosis, TdT can detect the DNA double-strand breaks with single base 3' overhangs by binding to exposed 3'-OH ends of DNA fragments. Apoptotic nuclei were identified *in situ *in the four groups investigated: fresh (control for viability), damaged (control for death), E2-treated and HBC-treated (control for E2 treatment; Figure 1A). In order to semi-quantify the TdT-positive nuclei, the apoptotic index in tissues from each group was calculated. Few apoptotic nuclei were found in E2- and HBC-treated tissues similar to fresh tissues, while damaged tissues showed an extensive level of apoptosis (*P *= 0.05 for damaged versus E2- and HBC-treated and fresh; Figure 1A and 1B).

### Metabolic activity of cultured tissues

**Figure 2:**
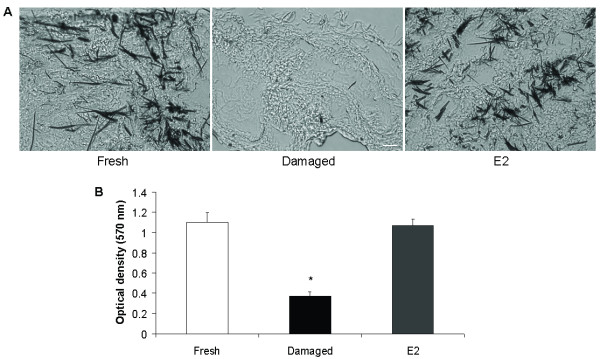
**Metabolic activity of cultured tissues**. (A) Representative images of 5 μm-thick tissue sections incubated in MTT. Fresh and E2-treated tissues display formazan crystal formation affirming tissue viability, while no crystals formed in damaged tissues. Scale bar, 50 μm. (B) Amount of dissolved formazan crystals measured in a spectrophotometer. In fresh and E2-treated tissues there are equally high amounts of crystals as determined by the absorbance measured, while in damaged tissues the crystal levels are significantly lower (*n *= 3, *P = 0.05 for damaged versus E2-treated and fresh). Data represent means ± standard deviation.

The MTT assay assesses the metabolic activity of cells and is a well-established method for determining cell viability. In viable and metabolically active cells, the mitochondrial dehydrogenase enzyme converts the soluble yellow MTT salt into insoluble purple formazan crystals. Determination of *in situ *formazan crystal formation in the different groups under investigation revealed that crystals formed both in fresh and E2-treated tissues but not in the group of damaged tissues (Figure 2A). Quantification of the metabolic activity confirmed that fresh and E2-treated tissues had very similar amounts of crystals while damaged tissues showed much lower crystal levels (*P *= 0.05 for damaged vs. E2-treated and fresh; Figure 2B). Therefore, E2-treated tissues retain their metabolic activity and remain viable, as demonstrated by the formation of formazan crystals.

### Sex-specific PGR regulation by E2

**Figure 3:**
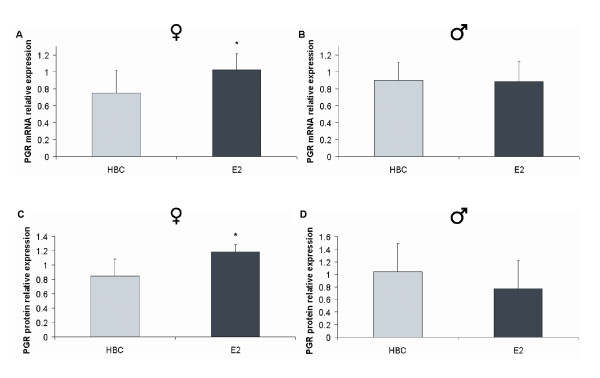
**Sex-specific *PGR *(progesterone receptor) regulation by E2 in human cardiac tissues**. (A) E2 increased the *PGR *gene expression level in tissues of female individuals (*n *= 4, adjusted *P *= 0.001) but (B) the same treatment had no effect in tissues from male individuals (*n *= 9, adjusted *P *= 0.9). (C) E2 exposure increased the PGR protein level in tissues of female individuals (*n *= 4, adjusted *P *= 0.03). (D) On the other hand, the same treatment with E2 revealed a decreasing trend of the PGR protein level in tissues of male individuals (*n *= 4, adjusted *P *= 0.079). Data represent means ± standard deviation.

Due to interests in hormonal effects and for the validation of the tissue culture system, human cardiac tissues were treated with E2 and the expression level of *PGR*, a known E2-target gene, was assessed by quantitative RT-PCR. It was discovered that the *PGR *mRNA level was modulated by E2 in a sex-specific manner as determined by analysis of variance (*n *= 13, interaction *P *< 0.001). *Post-hoc *analysis confirmed that following a 24-h E2-treatment the mRNA level of *PGR *displayed a 1.36-fold significant increase in tissues of female individuals (*n *= 4, adjusted *P *= 0.001; Figure 3A). However, E2-exposure had no effect on the *PGR *expression level in tissues from male individuals (*n *= 9, adjusted *P *= 0.9; Figure 3B).

Subsequently, the abundance level of the PGR protein in cardiac tissues treated with E2 was investigated. Of great importance, it was discovered that the PGR protein follows the same expression pattern as the *PGR *gene after E2-exposure showing a sex-specific regulation (*n *= 8, interaction *P *= 0.0028). In particular, tissues of female individuals treated with E2 exhibited a 1.40-fold increase in the protein level of PGR (*n *= 4, adjusted *P *= 0.03; Figure 3C), while E2-treated tissues of male individuals revealed a decreasing trend for PGR (*n *= 4, adjusted *P *= 0.079; Figure 3D).

## Discussion

Our novel finding is that, with the aid of a robust system for the culture of intact human heart tissue, we showed that in response to E2, cardiac tissues from women increase PGR mRNA expression and protein level, while cardiac tissues from men do not. We believe this report is the first demonstrating that E2 acts in the heart in a sex-specific manner. A modified KHB was used to maintain whole cardiac tissue viability. KHB with no further supplements has generally been used for the transport of explanted hearts [18,19] and for the short stimulation of myocardial strips [16] but not for the culture of tissues. Furthermore, freshly oxygenated buffer with known oxygen pressure was used, as uncontrolled oxygenation might lead to artifacts. An approximately 2-3 mm tissue thickness enabled oxygenation of the tissues by diffusion.

Moreover, two standard and well-received assays were recruited to assess the viability and the metabolic activity of the tissues. The TdT assay demonstrated extensive apoptosis in the group of damaged tissues (Figure 1A and 1B). The very few apoptotic nuclei observed in E2- and HBC-treated and in fresh tissues were expected, as some cell death would occur (Figure 1A and 1B). Similarly, the MTT assay revealed a maintained metabolic activity of the E2-treated tissues compared to fresh tissues used as positive controls for viability (Figure 2A and 2B). On the other hand, the negative controls (damaged tissues) showed very low levels of metabolic activity (Figure 2A and 2B). Both the TdT and MTT assays indicate that the current system maintains the human cardiac tissues in culture viable for at least 24 h.

When validating the system, it was found that the expression level of the *PGR *mRNA increases in the female human myocardium but does not change in the male human myocardium following E2 treatment (Figure 3A and 3B). The modest increase observed in *PGR *expression was expected, as small fold changes due to E2 treatment have been previously reported [20,21]. More importantly, a similar increase was identified at the protein level of PGR (Figure 3C and 3D). In addition, the heart is a strictly-regulated environment, where it is expected that minor changes have major effects. Indeed, the effects of progestins vary greatly and are tissue dependent. Both progesterone and its receptor are necessary for successful pregnancy and birth [22-24]. In observational studies, progesterone acts in a vasoconstrictive way [25-28], while it exerts vasorelaxation effects on vascular tissue *in vitro *[29]. In animal studies, progestins have been reported to reverse the atheroprotective effects of E2 [30-33]. Additionally, molecular studies have shown progesterone to regulate transcription in vascular endothelial cells and in vascular smooth muscle cells, where it inhibits proliferation through the modification of cyclin E and A transcription, which in turn leads to arrest in the G_0_-G_1 _stage [4,5,34,35].

Although, the regulation of *PGR *by E2 in several cell types has been well documented and the sex-specific manner of this has been described in the brain [8] and in human lung adenocarcinoma cell lines [9], to our knowledge, how E2 might affect the PGR gene and protein levels in the heart has not been previously reported. Furthermore, the mechanism responsible for this sex-specific regulation currently is not well understood. However, we believe that the recruitment of different cofactors to the ligand-receptor complex at the promoter region of *PGR *between the two sexes might be a crucial step. In addition, it has to be mentioned that the exact cellular type, where this regulation occurs, has not been identified. Nevertheless, in future studies this issue needs to be addressed.

The protective effects of E2 on the cardiovascular system have long been recognized and several biochemical pathways by which E2 could exert such effects have been described [1]. We propose further insight into how E2 might protect against cardiac remodelling. The proliferation of cardiac fibroblasts contributes to pathological structural changes in the heart [36]. E2 inhibits cardiac fibroblast growth [36]. Furthermore, in addition to the anti-proliferative effects of progesterone on vascular cells discussed above, progesterone inhibits DNA synthesis in neonatal cardiac fibroblasts [6], cardiac fibroblast growth and enhances the inhibitory effects of E2 [36]. Thus, it has been suggested that hormone therapy using E2 and progesterone may protect postmenopausal women against cardiovascular disease by inhibiting cardiac fibroblast growth and cardiac remodelling [36]. However, in the light of our results a similar therapy with E2 would not have the same potentially beneficial outcome in men.

Indeed, both men and women produce E2, although in different amounts, which also vary depending on age. We put forward that the observed induction in the PGR expression mediated by E2 in the female human myocardium facilitates the anti-proliferative effects of progesterone on cardiac fibroblasts through the E2-dependent increase of the responsiveness of these cells to progesterone; hence E2 modulates remodelling in the heart facilitating a beneficial outcome in women. Our hypothesis is in agreement with and may explain the observations made in human and animal studies, where females undergo more favourable remodelling than males. Along this line, we speculate that premenopausal women have higher cardiac PGR levels than both postmenopausal women and men due to higher endogenous E2 levels, hence providing an additional explanation for the later onset of cardiovascular disease observed generally in women.

## Conclusions

In conclusion, we have shown that E2 induces the expression of the PGR mRNA and protein in the myocardium of women but not in that of men. In addition, we have developed a hypothesis implicating this sex-specific regulation of PGR by E2 in the development of disease in men and women, which awaits further investigation. Furthermore, our biological discovery proves the usefulness and the relevance of the *ex vivo *system we have developed for tissue culture. We anticipate that it will allow further genomic and proteomic studies on a high-throughput scale offering insights and advancing our understanding of the cellular effects of stimuli and compounds that drive the tissue response to disease in humans.

## Abbreviations

ECM: extracellular matrix; HBC: 2-hydroxypropyl-β-cyclodextrin; KHB: Krebs Henseleit buffer; mRNA: messenger RNA; PBS: phosphate buffered saline; PGR: progesterone receptor; RT-PCR: real-time polymerase chain reaction; TdT: terminal deoxynucleotidyl transferase.

## Competing interests

The authors declare that they have no competing interests.

## Authors' contributions

GK designed and performed research, analysed and interpreted data, performed statistical analysis and wrote the manuscript. EB designed and supervised research and revised the manuscript. SM participated in the analysis of protein data. CK and RH planned and coordinated the selection of patients. VRZ supervised research and revised the manuscript. All authors read and approved the final manuscript.
